# Mitochondrial markers predict survival and progression in non-small cell lung cancer (NSCLC) patients: Use as companion diagnostics

**DOI:** 10.18632/oncotarget.19677

**Published:** 2017-07-28

**Authors:** Federica Sotgia, Michael P. Lisanti

**Affiliations:** ^1^ Translational Medicine, School of Environment & Life Sciences, University of Salford, Greater Manchester, United Kingdom

**Keywords:** lung cancer, mitochondrial biomarkers, treatment failure, relapse, recurrence

## Abstract

Here, we used an informatics-based approach to identify novel biomarkers of overall survival and tumor progression in non-small cell lung cancer (NSCLC) patients. We determined whether nuclear-encoded genes associated with mitochondrial biogenesis and function can be used to effectively predict clinical outcome in lung cancer. This strategy allowed us to directly provide *in silico* validation of the prognostic value of these mitochondrial components in large, clinically-relevant, lung cancer patient populations. Towards this end, we used a group of 726 lung cancer patients, with negative surgical margins. Importantly, in this group of cancer patients, markers of cell proliferation (Ki67 and PCNA) were associated with poor overall survival, as would be expected. Similarly, key markers of inflammation (CD163 and CD68) also predicted poor clinical outcome in this patient population. Using this approach, we identified >180 new individual mitochondrial gene probes that effectively predicted significantly reduced overall survival, with hazard-ratios (HR) of up to 4.89 (p<1.0e-16). These nuclear-encoded mitochondrial genes included chaperones, membrane proteins as well as ribosomal proteins (MRPs) and components of the OXPHOS (I-V) complexes. In this analysis, HSPD1, a key marker of mitochondrial biogenesis, had the highest predictive value and was also effective in predicting tumor progression in both smokers and non-smokers alike. In fact, it had even higher predictive value in non-smokers (HR=5.9; p=3.9e-07). Based on this analysis, we conclude that mitochondrial biogenesis should be considered as a new therapeutic target, for the more effective treatment of human lung cancers. The mitochondrial biomarkers that we have identified could serve as new companion diagnostics to assist clinicians in more accurately predicting clinical outcomes in lung cancer patients, driving more personalized cancer therapy.

## INTRODUCTION

Treatment failure is the most critical obstacle for more effective anti-cancer therapy and personalized medicine [1, 2]. As such, this still dramatically limits the efficacy of most cancer treatments, especially in lung cancer patients. As a consequence, better biomarkers are needed for the early stratification of lung cancer patients into low-risk and high-risk groups at diagnosis [1–3].

Here, we examined the hypothesis that markers of mitochondrial biogenesis and function may have significant prognostic value in the early identification of high-risk lung cancer patients, with poor overall clinical survival and tumor progression. In this context, we employed a bioinformatics approach to assess the possible utility of nuclear-encoded mitochondrial gene transcripts in predicting clinical outcome.

Our results indicate that > 180 different mitochondrial gene probes can be used individually, to predict poor overall survival in lung cancer patients. As such, we discuss the possibility that mitochondria should be therapeutically targeted, to improve the effectiveness of current lung cancer therapy and overall survival.

## RESULTS

### Value of proliferative and inflammatory markers in the patient population

**Figure 1:**
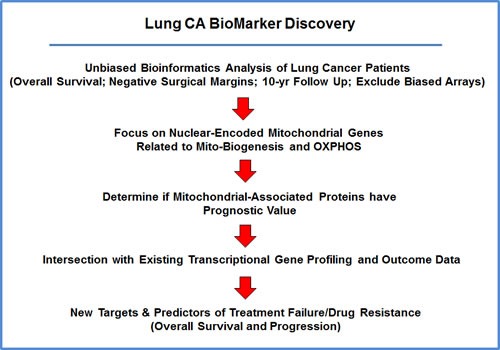
Diagram showing our bio-informatics approach to lung cancer biomarker discovery For this analysis, we chose to focus on non-small lung cancer patients, with negative surgical margins, and 10-years of follow-up data (*N* = 726). In this context, we evaluated the prognostic value of mitochondrial markers for predicting overall survival, time to first progression, and post-progression survival.

**Figure 2:**
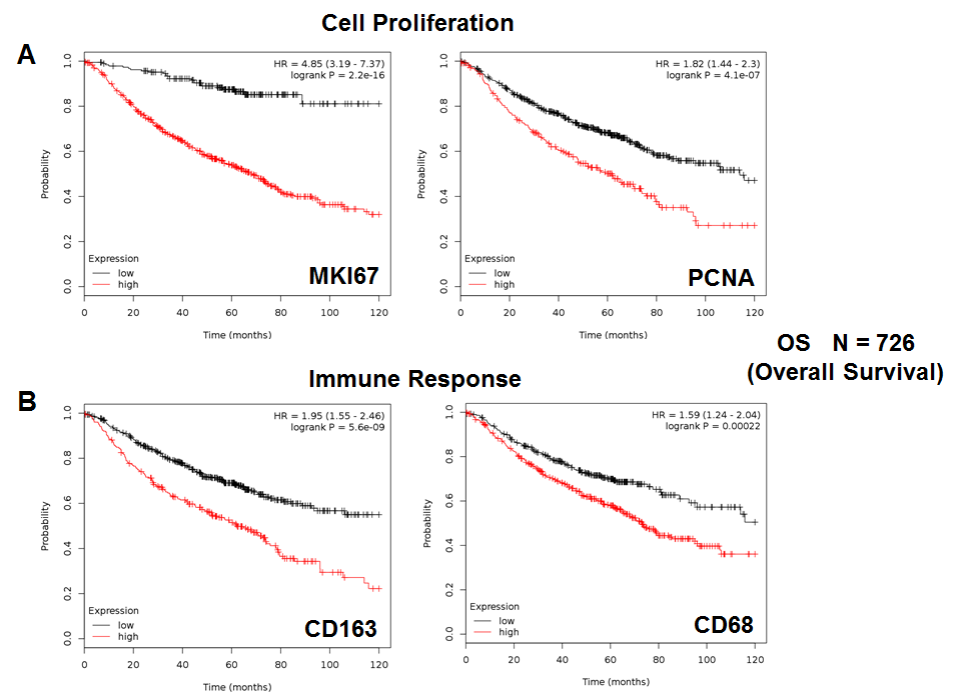
Markers of proliferation and inflammation predict poor overall survival in high-risk lung cancer patients We assessed the predictive value of Ki67 and PCNA in *N* = 726 lung cancer patients, with negative surgical margins. **A.** Note that high transcript levels of Ki67 and PCNA are associated with significantly reduced overall survival. Please note that the official gene name for the Ki67 protein is MKI67. **B.** Note that that high transcript levels of CD163 and CD68 are associated with significantly reduced overall survival.

To identify new potential biomarkers, here we used publically available transcriptional profiling data from the tumors of lung cancer patients, with negative surgical margins (Figure 1), with 10 years of follow-up. Since proliferative markers are used as primary endpoints in clinical trials, we first assessed the prognostic value of Ki67 and PCNA, in this patient population. Tables 1, 2 and Figure 2A both show the prognostic value of these markers. The hazard-ratios for Ki67 and PCNA were 4.85 and 1.82, respectively, for overall survival (OS).

**Table 1:** Prognostic Value of KI67 in Lung Cancer

Gene Probe ID	Symbol	Hazard-Ratio	Log-Rank Test
212020_s_at	MKI67	4.85	2.2e-16
212021_s_at	MKI67	3.11	3.4e-11
212023_s_at	MKI67	3.04	2.4e-12
212022_s_at	MKI67	2.96	7.4e-14
**Combined**		**4.43**	**7.0e-14**

**Table 2:** Prognostic Value of PCNA and Markers of Inflammation in Lung Cancer

Gene Probe ID	Symbol	Hazard-Ratio	Log-Rank Test
217400_at	PCNA	1.82	4.1e-07
216233_at	CD163	1.95	5.6e-09
215049_x_at	CD163	1.39	0.006
203645_s_at	CD163	1.30	0.03
203507_at	CD68	1.59	0.0002

We also assessed the prognostic value of two macrophage-specific markers of inflammation. Table 2 and Figure 2B show that CD163 and CD68 both effectively predict overall survival, with hazard-ratios of 1.95 and 1.59, respectively. Thus, conventional markers of proliferation and inflammation can be used to predict overall survival in lung cancer patients.

### Value of individual mitochondrial markers

To test our hypothesis that increased mitochondrial mass, biogenesis and function contributes towards poor overall survival in lung cancer patients, we next assessed the prognostic value of specific mitochondrial markers.

**Figure 3:**
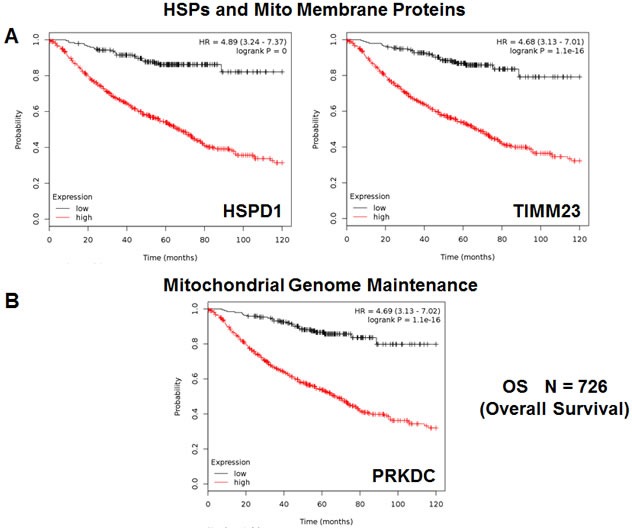
HSPD1, mitochondrial membrane proteins and PRKDC are associated with poor clinical outcome in lung cancer patients **A.** Note that that high transcript levels of HSPD1 and TIMM23 are associated with significantly reduced overall survival. **B.** Note that that high transcript levels of PRKDC are associated with significantly reduced overall survival.

Initially, we examined the behavior of mitochondrial chaperones and mitochondrial membrane proteins. Table 3 and Figure 3 both show that HSP60 (HSPD1) has the best prognostic value, with a hazard-ratio of 4.89 (*p* < 1.0e-17). Members of the TIMM and TOMM gene families also had prognostic value; AKAP1 and SLC25A5 also had significant value. Similar results were also obtained with mitochondrial creatine kinase isoforms (HR = 2.88-to-1.51) and PRKDC (DNA-PK), a critical kinase that helps maintain the integrity and the copy number of the mitochondrial genome (mt-DNA) (HR = 4.69-to-1.65), which functions in the DNA damage response.

**Table 3:** Prognostic Value of Mitochondrial HSPs and Other Mitochondrial Proteins

Gene Probe ID	Symbol	Hazard-Ratio	Log-Rank Test
**HSPs and Membrane Proteins (28 probes in total)**			
200806_s_at	HSPD1	4.89	<1.0e-16
218119_at	TIMM23	4.68	1.1e-16
218357_s_at	TIMM8B	4.26	7.8e-16
203342_at	TIMM17B	3.31	2.5e-11
203093_s_at	TIMM44	2.29	1.1e-09
217981_s_at	TIMM10B	2.15	1.2e-06
218316_at	TIMM9	2.06	4.3e-08
201821_s_at	TIMM17A	2.04	1.7e-09
218188_s_at	TIMM13	1.94	8.5e-09
218118_s_at	TIMM23	1.83	1.8e-07
218408_at	TIMM10	1.79	4e-05
202264_s_at	TOMM40	4.29	1.1e-14
217960_s_at	TOMM22	3.19	1.3e-13
201870_at	TOMM34	2.83	9.8e-12
201812_s_at	TOMM7	2.84	5.4e-13
201512_s_at	TOMM70A	1.90	3.1e-08
212773_s_at	TOMM20	1.54	0.0006
217139_at	VDAC1	3.74	1.9e-14
217140_s_at	VDAC1	2.58	1.1e-16
212038_s_at	VDAC1	1.63	7.8e-05
208844_at	VDAC3	3.64	3.9e-14
211662_s_at	VDAC2	2.36	6e-14
210625_s_at	AKAP1	1.88	1.3e-06
200657_at	SLC25A5	1.54	0.0001
**Mitochondrial Creatine Kinase (2 probes in total)**			
202712_s_at	CKMT1A	2.88	7.8e-10
205295_at	CKMT2	1.51	0.0005
**Mitochondrial Genome Maintenance (3 probes in total)**			
210543_s_at	PRKDC	4.69	1.1e-16
208694_at	PRKDC	2.23	4.3e-12
215757_at	PRKDC	1.65	4.0e-05

**Figure 4:**
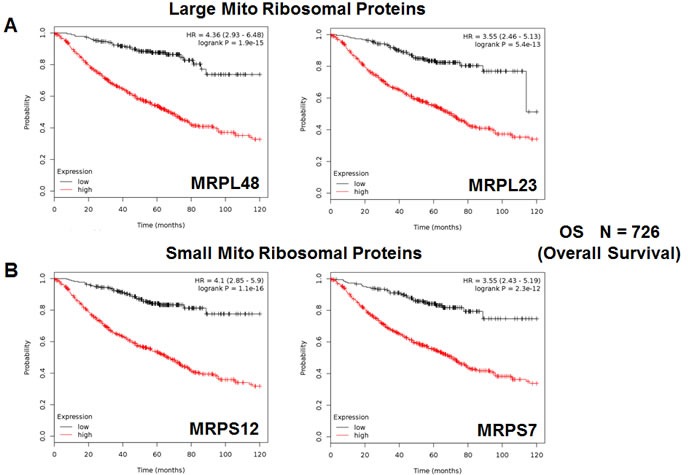
Mitochondrial ribosomal proteins (MRPs) are associated with poor clinical outcome in lung cancer patients **A.** Note that high transcript levels of MRPL48 and MRPL23 predict significantly reduced overall survival. **B.** Similarly, high transcript levels of MRPS12 and MRPS7 predict significantly reduced overall survival.

Secondly, we examined the prognostic value of mitochondrial ribosomal proteins (MRPs), which contribute to the synthesis of key members of the OXPHOS-complexes, and are essential for mitochondrial biogenesis (Table 4). Twenty-one components of the large subunit (MRPLs) showed significant prognostic value, with hazard-ratios between 4.36 and 1.47. Notably, MRPL48 had the best prognostic value. Fifteen different components of the small subunit (MRPSs) showed significant prognostic value, with hazard-ratios between 4.10 and 1.27. As such, thirty-six different MRPs all predicted poor overall survival. Kaplan-Meier curves for representative examples are shown in Figure 4, panels A & B.

**Table 4:** Prognostic Value of Mitochondrial Ribosomal Proteins

Gene Probe ID	Symbol	Hazard-Ratio	Log-Rank Test
**Large Ribosomal Subunit (21 probes in total)**			
218281_at	MRPL48	4.36	1.9e-15
213897_s_at	MRPL23	3.55	5.4e-13
219162_s_at	MRPL11	3.29	2.5e-13
221997_s_at	MRPL52	3.20	3.6e-14
221692_s_at	MRPL34	3.08	1.6e-11
203931_s_at	MRPL12	2.82	3.3e-12
218887_at	MRPL2	2.81	4.4e-11
217919_s_at	MRPL42	2.54	1.6e-13
218270_at	MRPL24	2.35	1.8e-09
218105_s_at	MRPL4	2.32	1.6e-09
218202_x_at	MRPL44	2.19	2.5e-10
222216_s_at	MRPL17	2.02	1.4e-08
218890_x_at	MRPL35	1.96	5.7e-09
204599_s_at	MRPL28	1.91	1.4e-07
220527_at	MRPL20	1.84	9.1e-05
201717_at	MRPL49	1.68	8.7e-06
218049_s_at	MRPL13	1.68	8.1e-06
217980_s_at	MRPL16	1.66	1.5e-05
203152_at	MRPL40	1.62	0.0001
218027_at	MRPL15	1.59	0.0001
203781_at	MRPL33	1.47	0.001
**Small Ribosomal Subunit (19 probes in total)**			
204331_s_at	MRPS12	4.10	1.1e-16
210008_s_at	MRPS12	3.93	4.9e-14
204330_s_at	MRPS12	3.27	1e-13
213840_s_at	MRPS12	2.99	2.3e-12
217932_at	MRPS7	3.55	2.3e-12
218001_at	MRPS2	3.28	1e-11
221688_s_at	MRPS4	3.09	7.7e-11
211595_s_at	MRPS11	2.96	9.1e-12
215919_s_at	MRPS11	1.55	0.0002
218112_at	MRPS34	2.43	7.6e-08
212604_at	MRPS31	2.29	2.7e-07
219819_s_at	MRPS28	1.74	2.7e-06
217942_at	MRPS35	1.70	8.4e-06
221437_s_at	MRPS15	1.59	0.0001
12145_at	MRPS27	1.61	7.4e-05
218398_at	MRPS30	1.47	0.003
218654_s_at	MRPS33	1.35	0.01
203800_s_at	MRPS14	1.27	0.05

**Figure 5:**
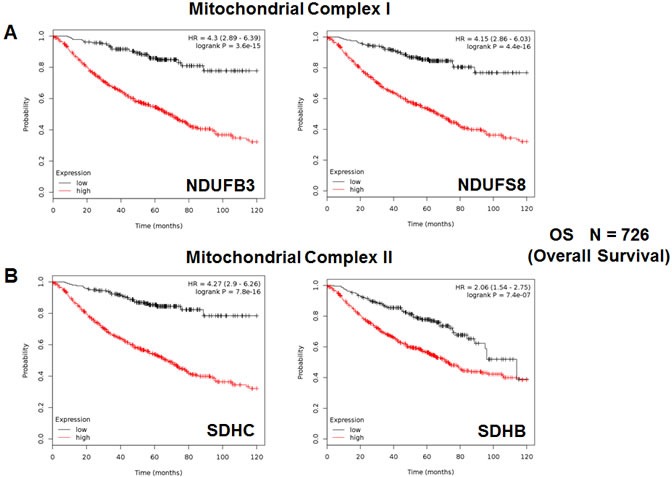
Mitochondrial complex I and II proteins are associated with poor clinical outcome in lung cancer patients **A.** Note that high levels of NDUFB3 and NDUFS8 predict significantly reduced overall survival. **B.** Similarly, high levels of SDHC and SDHB predict significantly reduced overall survival.

**Figure 6:**
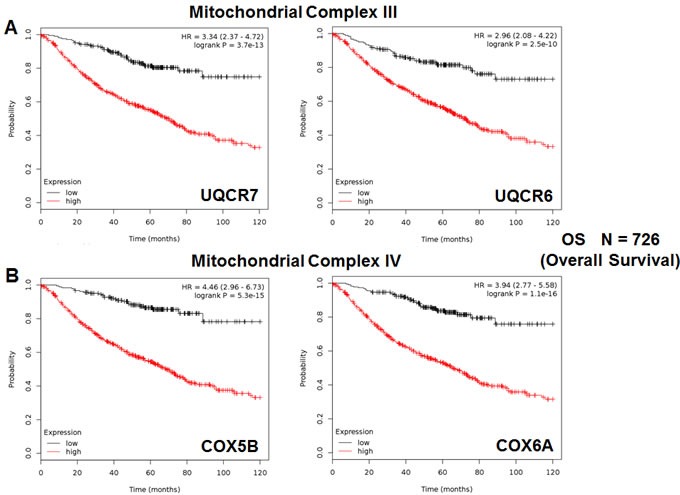
Mitochondrial complex III and IV proteins are associated with poor clinical outcome in lung cancer patients **A.** Note that high levels of UQCR7 and UQCR6 predict significantly reduced overall survival. **B.** Similarly, high levels of COX5B and COX6A predict significantly reduced overall survival.

**Figure 7:**
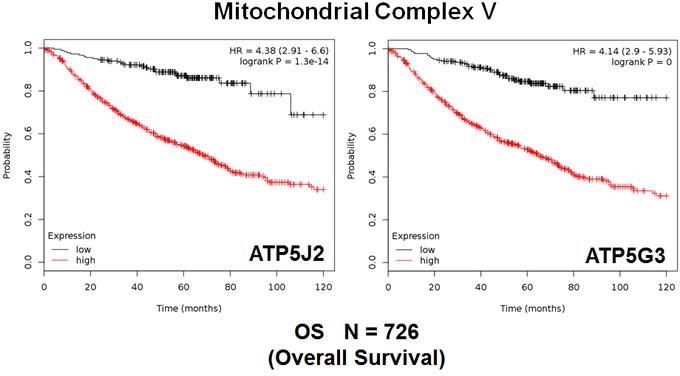
Mitochondrial complex V proteins are associated with poor clinical outcome in lung cancer patients Note that high levels of ATP5J2 and ATP5G3 predict significantly reduced overall survival.

We also assessed the prognostic value of members of the OXPHOS complexes I-V. These results are summarized in Table 5. Remarkably, 88 different gene probes for the OXPHOS complexes showed hazard-ratios between 4.46 and 1.39. COX5B (complex IV) had the best prognostic value (HR = 4.46; *p* = 5.3e-15). NDUFB3 (complex I) also showed significant prognostic value (HR = 4.30; *p* = 3.6e-15). Kaplan-Meier curves for members of complex I and II are shown in Figure 5A & 5B, while results with members of complex III and IV are shown in Figure 6A & 6B. Results with complex V are shown in Figure 7.

**Table 5:** Prognostic Value of Mitochondrial OXPHOS Complexes

Gene Probe ID	Symbol	Hazard-Ratio	Log-Rank Test
**Complex I (27 probes in total)**			
203371_s_at	NDUFB3	4.30	3.6e-15
203189_s_at	NDUFS8	4.15	4.4e-16
203190_at	NDUFS8	2.94	2.1e-11
209303_at	NDUFS4	3.83	1.1e-15
218484_at	NDUFA4L2	3.33	2.1e-13
218226_s_at	NDUFB4	3.21	1.8e-14
220864_s_at	NDUFA13	3.00	9.5e-11
202941_at	NDUFV2	3.00	1.3e-13
201740_at	NDUFS3	2.92	1.2e-11
217860_at	NDUFA10	2.77	3e-14
218563_at	NDUFA3	2.23	1.9e-10
214241_at	NDUFB8	2.23	1.5e-09
218201_at	NDUFB2	2.21	1.2e-08
215850_s_at	NDUFA5	1.83	3.6e-07
202785_at	NDUFA7	1.81	3e-07
202298_at	NDUFA1	1.72	3e-06
201966_at	NDUFS2	1.70	6.6e-06
202839_s_at	NDUFB7	1.64	0.0009
201757_at	NDUFS5	1.64	4.3e-05
209224_s_at	NDUFA2	1.59	6.6e-05
208969_at	NDUFA9	1.56	0.0002
211752_s_at	NDUFS7	1.50	0.0007
203613_s_at	NDUFB6	1.49	0.0009
209223_at	NDUFA2	1.49	0.0009
218320_s_at	NDUFB11	1.48	0.001
218200_s_at	NDUFB2	1.48	0.001
208714_at	NDUFV1	1.44	0.002
**Complex II (5 probes in total)**			
216591_s_at	SDHC	4.27	7.8e-16
202004_x_at	SDHC	3.64	4e-14
210131_x_at	SDHC	3.45	4.2e-14
202675_at	SDHB	2.06	7.4e-07
214166_at	SDHB	1.94	2.5e-08
**Complex III (8 probes in total)**			
201568_at	UQCR7	3.34	3.7e-13
209066_x_at	UQCR6	2.96	2.5e-10
202233_s_at	UQCR8	2.09	5.9e-07
208909_at	UQCRFS1	1.69	2.6e-05
201066_at	UQCR4/CYC1	1.54	0.0006
207618_s_at	BCS1L	1.54	0.0003
205849_s_at	UQCR6	1.48	0.0008
202090_s_at	UQCR	1.45	0.004
**Complex IV (19 probes in total)**			
211025_x_at	COX5B	4.46	5.3e-15
202343_x_at	COX5B	3.97	1.1e-16
213735_s_at	COX5B	2.15	9.6e-10
213736_at	COX5B	1.51	0.0015
200925_at	COX6A	3.94	1.1e-16
201119_s_at	COX8A	3.78	2.4e-15
203880_at	COX17	3.55	3.9e-15
201754_at	COX6C	3.24	1.8e-14
217249_x_at	COX7A2	3.05	3.3e-13
201441_at	COX6B	2.93	3.8e-12
206353_at	COX6A2	2.77	1.8e-11
203858_s_at	COX10	2.44	1.3e-09
202110_at	COX7B	2.29	2.5e-12
216003_at	COX10	2.18	1.8e-07
221550_at	COX15	2.09	1.5e-10
217451_at	COX5A	2.01	9e-06
218057_x_at	COX4NB	1.54	0.0008
204570_at	COX7A	1.51	0.0015
202698_x_at	COX4I1	1.39	0.01
**Complex V (23 probes in total)**			
202961_s_at	ATP5J2	4.38	1.3e-14
207507_s_at	ATP5G3	4.14	<1e-17
207508_at	ATP5G3	2.34	1.6e-13
210149_s_at	ATP5H	3.70	3.7e-15
209492_x_at	ATP5I	3.33	7.7e-13
207335_x_at	ATP5I	2.14	2e-08
203926_x_at	ATP5D	3.02	2.7e-11
213041_s_at	ATP5D	2.41	3.1e-10
208764_s_at	ATP5G2	2.75	2.9e-10
207552_at	ATP5G2	2.55	4.3e-09
217368_at	ATP5G2	1.85	4.9e-07
217801_at	ATP5E	2.62	2e-09
210453_x_at	ATP5L	2.56	1.8e-11
207573_x_at	ATP5L	2.25	1.9e-10
208746_x_at	ATP5L	2.10	7.4e-10
201322_at	ATP5B	1.88	1.5e-07
206992_s_at	ATP5S	1.88	2.9e-07
206993_at	ATP5S	1.85	2.1e-07
208972_s_at	ATP5G	1.87	5.4e-08
221677_s_at	ATP5O	1.71	6.8e-06
208870_x_at	ATP5C	1.54	0.0008
205711_x_at	ATP5C	1.42	0.004
213366_x_at	ATP5C	1.40	0.007

### Mitochondrial genes have predictive value in both “smoking” and “non-smoking” patient populations: overall survival and tumor progression

**Figure 8:**
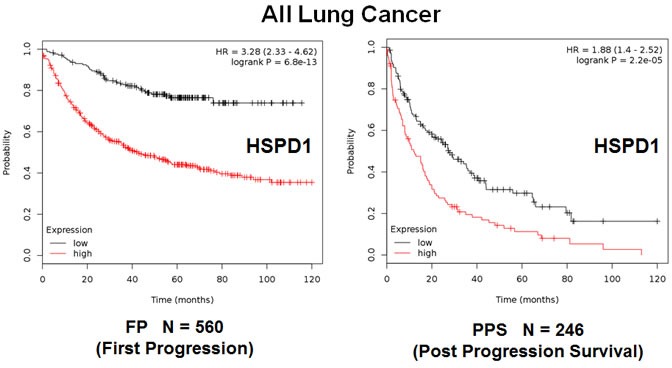
The mitochondrial chaperone, HSPD1, predicts tumor progression in lung cancer patients Note that the levels HSPD1 effectively predict time to first progression (Left panel) and post-progression survival (Right panel).

In order to further test the prognostic power of these individual mitochondrial biomarkers, we next selected the most promising one, HSPD1, and assessed its ability to predict tumor progression in the whole patient population (*N* = 726). Importantly, Figure 8 shows that the levels of HSPD1 effectively predict time to tumor progression and post-progression survival, with hazard ratios of 3.28 and 1.88, respectively.

**Figure 9:**
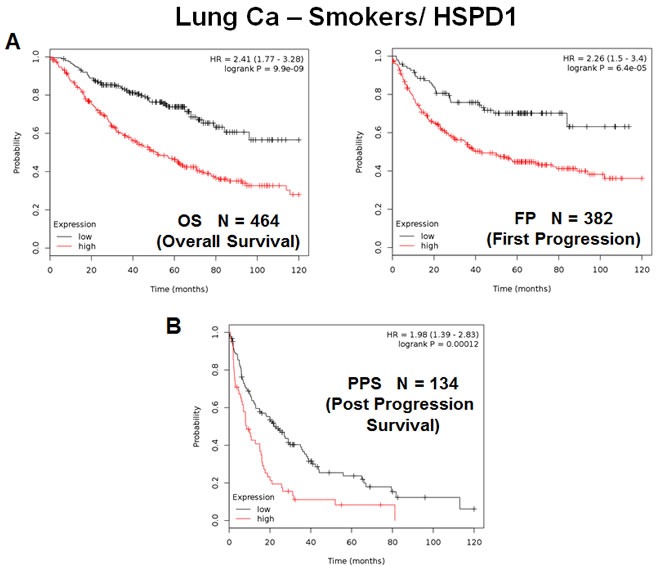
The mitochondrial chaperone, HSPD1, predicts poor clinical outcome and tumor progression in lung cancer patients: Smokers Note that the levels HSPD1 effectively predict overall survival **A.**, as well as time to first progression and post-progression survival **B.**, in the “smoking” patient population.

**Figure 10:**
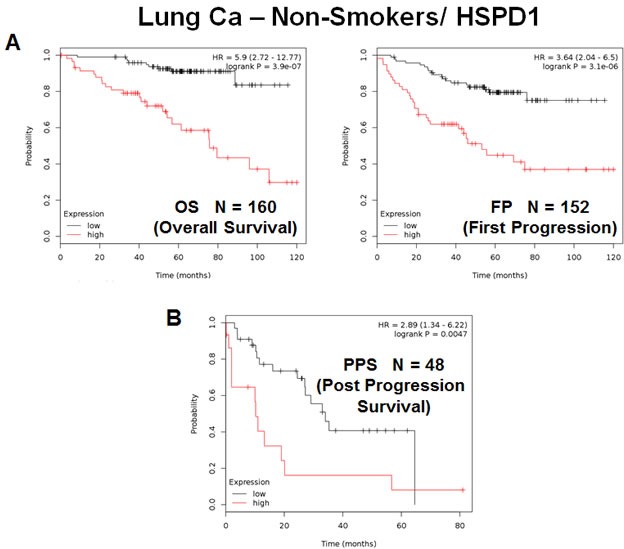
The mitochondrial chaperone, HSPD1, predicts poor clinical outcome and tumor progression in lung cancer patients: Non-Smokers Note that the levels HSPD1 effectively predict overall survival **A.**, as well as time to first progression and post-progression survival **B.**, in the “non-smoking” patient population.

A similar analysis was also carried out when the patient population was sub-divided into smokers (*N* = 464) and non-smokers (*N* = 160) (Figures 9 and 10). Using this approach, HSPD1 showed increased prognostic power in the non-smoking patient population, reaching a hazard-ratio of 5.9 for overall survival; however, HSPD1 still retained its prognostic value in the smoking patient population (Figures 9A and 10A).

In this context, this trend was also true for tumor progression, as HSPD1 was a better predictor of time to tumor progression and post-progression survival in non-smokers (Figures 9B and 10B), with hazard-ratios of 3.64 and 2.89, respectively.

Thus, the mitochondrial chaperone, HSPD1, is an effective predictive biomarker of overall survival and tumor progression, in both smokers and non-smokers as well.

## DISCUSSION

### Linking CSC propagation with telomerase activity and mitochondrial function: Targeting CSCs with doxycycline and/or palbociclib

Recently, we determined the functional role of telomerase activity in lung cancer stem cell (CSC) propagation. More specifically, we indirectly monitored telomerase activity, by linking the hTERT-promoter to eGFP [4, 5]. Using A549 lung cancer cells, stably-transfected with the hTERT-GFP reporter, we then used GFP-expression fluorescence intensity to fractionate these cell lines into GFP-high and GFP-low cell populations. We functionally compared the phenotype of these GFP-high and GFP-low cell sub-populations. Importantly, we directly demonstrated that cancer cells with higher telomerase activity (GFP-high) are energetically-activated, with increased mitochondrial function and increased glycolysis. This was directly confirmed by proteomics analysis. Cells with high telomerase activity showed increased stem cell activity (measured *via* 3D-spheroid formation) and an increased capacity for cell migration (measured with a Boyden-chamber). These phenotypes were blocked by inhibitors of energy-metabolism, which targeted either mitochondrial OXPHOS or glycolysis, or by using doxycycline, an FDA-approved antibiotic, that inhibits mitochondrial biogenesis as an off-target effect [4, 5].

The levels of telomerase activity also determined the ability of hTERT-high CSCs to proliferate, as assessed by measuring DNA synthesis [4, 5]. Treatment with Palbociclib, an FDA-approved CDK4/6 inhibitor specifically blocked the propagation of lung CSCs, at concentrations in the nanomolar range. Therefore, telomerase-high CSCs are among the most energetically activated, migratory and proliferative cell sub-populations. These observations may provide a mechanistic explanation for why long telomere length [6–9] (a surrogate marker of increased telomerase activity) is specifically associated with metastasis and poor clinical outcome in NSC lung cancer and many other tumor types. Thus, high telomerase activity may drive poor clinical outcome by activating mitochondrial biogenesis, “fueling” the proliferation in lung CSCs [4, 5].

### Using mitochondrial markers as companion diagnostics in NSCLC patients: Importance for treatment stratification and personalized medicine

Consistent with this novel hypothesis linking high telomerase activity with enhanced mitochondrial function, we show here that mitochondrial markers effectively predict poor overall survival in lung cancer patients, with negative surgical margins. Importantly, these mitochondrial markers could now be used to identify high-risk lung cancer patients at diagnosis, up to 10 years in advance. These results also suggest that mitochondria should be therapeutically-targeted in epithelial lung cancer cells to significantly extend patient survival.

**Figure 11:**
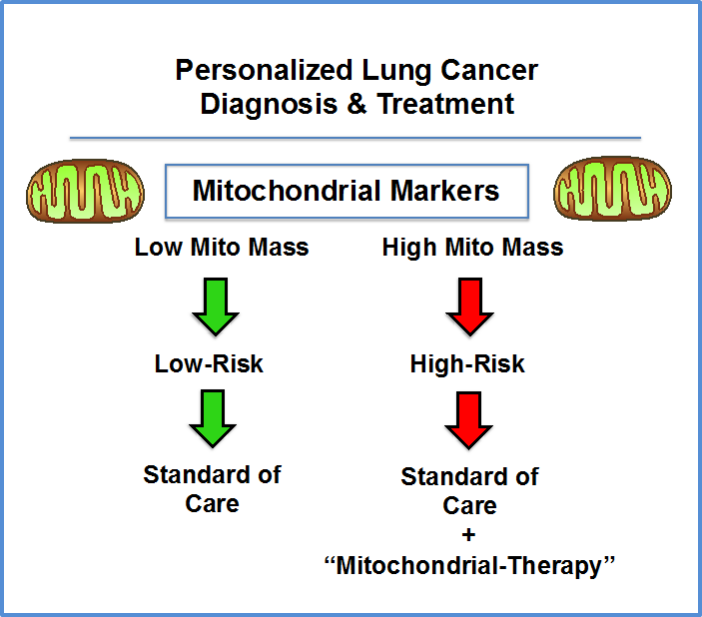
NSC lung cancer: mitochondrial-based diagnostics for personalized cancer therapy In this diagram, mitochondrial-based diagnostics would be used to separate lung cancer patients into high-risk and low-risk groups. Then, patients with high levels of mitochondrial markers in their primary tumor (“bad prognosis”) would be treated with mitochondrial-based therapies (such as “Doxycycline”), as an add-on to the standard of care, to prevent tumor progression and increase overall survival.

In this workflow, high-risk patients should be first identified at diagnosis by the high expression of mitochondrial markers in their primary lung tumors (Figure 11). Then, these patients could be treated with FDA-approved therapeutics (e.g., Doxycycline or Palbociclib; in combination with the standard of care), to improve poor overall survival. Importantly, both of these drugs have already been shown to be effective against the propagation of the lung CSC sub-population.

In this context, these mitochondrial markers could also be used as effective companion diagnostics for new experimental therapeutics targeting either mitochondria or telomerase (hTERT) and/or cell proliferation, to select the high-risk lung cancer patient sub-group, allowing proper treatment stratification.

## MATERIALS AND METHODS

### Kaplan-Meier (K-M) analyses

To perform K-M analysis on nuclear mitochondrial gene transcripts, we used an open-access online survival analysis tool to interrogate publically available microarray data from up to 1,926 lung cancer patients [3]. This allowed us to determine their overall prognostic value. For this purpose, we primarily analyzed 10-year follow-up data from non-small cell lung cancer (NSCLC) patients that had negative surgical margins (*N* = 726) [3]. Biased array data were excluded from the analysis. This allowed us to identify > 180 nuclear mitochondrial gene probes, with significant prognostic value. Hazard-ratios were calculated, at the best auto-selected cut-off, and p-values were calculated using the logrank test and plotted in R. K-M curves were also generated online using the K-M-plotter (as high-resolution TIFF files), using univariate analysis:

http://kmplot.com/analysis/index.php?p = service&cancer = lung.

This allowed us to directly perform *in silico* validation of these mitochondrial biomarker candidates. The most updated version of the database (2015) was utilized for all these analyses.

## Abbreviations

CSCs: cancer stem-like cells
FP: first progression
HR: hazard ratio
K-M: Kaplan-Meier
LN: lymph node
MRPL: mitochondrial ribosomal proteins, large subunit
MRPS: mitochondrial ribosomal proteins, small subunit
N: number of patients in a given data set
NSCLC: non-small cell lung cancer
OS: overall survival
OXPHOS: oxidative phosphorylation (mitochondrial respiration)
PPS: post progression survival
